# Physical Unclonable Function Based on the Internal State Transitions of a Fibonacci Ring Oscillator

**DOI:** 10.3390/s21113920

**Published:** 2021-06-07

**Authors:** Łukasz Matuszewski, Jakub Nikonowicz, Paweł Kubczak, Wiktor Woźniak

**Affiliations:** Faculty of Computing and Telecommunications, Poznań University of Technology, 60-965 Poznań, Poland; pawel.kubczak@put.poznan.pl (P.K.); wiktor.ma.wozniak@doctorate.put.poznan.pl (W.W.)

**Keywords:** device authentication, pattern matching, physical unclonable function, random number generator, ring oscillator

## Abstract

This article introduces a new class of physical unclonable functions (PUFs) based on the Fibonacci ring oscillator (FIRO). The research conducted here proves that before reaching the desired randomness, the oscillator shows a certain degree of repeatability and uniqueness in the initial sequence of internal state transitions. The use of an FIRO in conjunction with the restart method makes it possible to obtain a set of short boot sequences, which are processed with an innovative feature extraction algorithm that enables reliable device identification. This approach ensures the reuse of the existing random number generator (RNG), rather than multiplying ring oscillators in a dedicated structure. Moreover, the algorithm for the recovery of the device key from the boot set can be successfully implemented in the authorizing center, thus significantly releasing the resources of authorized low-complexity devices. The proposed methodology provides an easily obtainable key with identifiability, which was proven experimentally on FPGAs from different manufacturers.

## 1. Introduction

Authentication, authorization, and privacy are three sides of a security triangle that should be supported by every network device. Currently, due to the rapidly developing Internet of Things (IoT), ensuring cybersecurity at a high level is of particular importance. In typical cryptographic applications, secret keys are stored in either non-volatile or volatile memory. In the first case, they remain in memory and can be recovered even after powering off the device. In the latter, memory is vulnerable to attack if an adversary gains physical access to it. Therefore, traditional key storage approaches are not preferred and are slowly being replaced. Instead, a physical unclonable function (PUF) has been proposed as a lightweight, cost-effective, and ubiquitous solution. Generally, a PUF can be defined as an inherent and non-clonable feature specific to a given physical object, and it is used to generate a secure key. Unclonable functions are a promising solution that can be used wherever authentication, security, and key storage are needed without requiring secure memories or other expensive security hardware. Because PUFs promise to achieve secure authentication without any cryptographic resources on the device, they are of particular interest for resource-constrained IoT devices [1].

In recent years, physical unclonable functions have become the subject of intense research in both academic and industrial communities, and PUF-based solutions have evolved into essential components of modern secure systems for identification, key generation, and storage [1,2]. Depending on the specific manufacturing variances of a device, a PUF can be derived from several sources of randomness. Typical approaches refer to the variations in the manufacturing of wires and gates of CMOS circuits [1,2,3,4], e.g., by comparing delay paths of equal length in an arbiter PUF or by exploiting differences between frequencies of multiple ring oscillators (ROs) of the same structure—RO PUF. Other solutions rely on the inherent properties of memory cells in a digital circuit [1,4]—so called memory PUFs; these include, e.g., butterfly PUFs, which consist of two cross-coupled data latches with a clear/preset input that drives instability, SRAM PUF cells, which are composed of two cross-coupled inverters, and latch PUFs, which consist of cross-coupled NOR gates.

PUFs can be distinguished not only according to the type of their physical structure, but also through a safety classification. Categorization is based on the number of possible challenges in triggering secure responses and the external availability of the responses. A common category is the weak PUF, which provides a limited number of challenge–response pairs, as opposed to the strong PUF, for which the set of such pairs is much wider. Weak PUFs are the least susceptible to modeling attacks; hence, they are mainly used to obtain a secret key in cryptographic algorithms. Therefore, they are also known as physically obfuscated keys (POKs) [5], in which the secret key is permanently stored in the unique inaccuracy of the physical structure rather than being stored digitally, which makes it difficult for an opponent to learn the key through a probing attack [6].

In this article, we propose and study the possibility of using a novel class of RO-PUFs based on the response of a single Fibonacci ring oscillator (FIRO) that was proposed by Golić in [7]. We prove that, before achieving a desired randomness through jitter accumulation, each oscillator shows some degree of repetitiveness in an initial sequence of internal state transitions. Due to the inaccuracies in the technological process, the initial sequence remains unique for each device, ensuring high differentiation between circuits. The use of an FIRO in combination with a restart method [8] allows us to obtain a set of short boot sequences, which, when processed with an innovative algorithm for common feature extraction, allow the recovery of the key that identifies the source oscillator. The proposed methodology provides an easily obtainable RO PUF with experimentally proven identifiability.

The remainder of this paper is organized as follows. In Section 2, we review the related research in the field of physical unclonable functions based on ring oscillators. Section 3 describes the proposed device identification algorithm. Section 4 explains the experimental methodology and shows numerical results. Finally, Section 5 gives the concluding remarks.

## 2. Relevant Work

Due to their ease of deployment, RO PUFs are often the preferred form of FPGA-based solutions [9]. In these PUFs, the differences between frequencies of multiple ring oscillators of the same structure are typically used to generate a unique PUF response [9,10]. In prior studies, several approaches based on ring oscillators have been proposed and have achieved success in the generation of secure keys. The basic structure, which was first proposed in [11], compares oscillators’ output frequencies by counting the cycles in a certain time period. The proposed solution, which has been intensively developed over the last few years, has evolved into a whole group of solutions based on ring oscillators. Commonly used derivatives include:

- CRO PUF—the architecture is similar to that of the original RO PUF, but the RO section is replaced with a configurable ring oscillator (CRO) design. In this approach, only two ROs are used instead of a group of oscillators, and the configuration is set by driving multiplexers (MUX 2:1) inserted between each stage of the inverters. The MUX selection lines are configured as a challenge. Only a specific pair with maximum frequency separation is selected from the CRO challenge group to generate the response. Their frequency is measured by using a counter, and a comparator block is used in order to produce the output bit. The above scheme results in a lower number of ROs remaining unused during signature extraction than in the original case [12].
- TERO PUF—This is made of transient-effect ring oscillator (TERO) cells that have two states: an unstable state, which is called a transient oscillating state, and a stable state. This structure is similar to that of an RO PUF, but instead of using ring oscillators, TERO cells built on RS flip-flops are used. By setting the trigger signal to a high level, the circuit goes into an oscillation state for a very short period of time. The TERO cells are connected to two counters, and the signal passing through two multiplexers drives the correct output to the clock input of the counters. The TERO PUF is not as susceptible to frequency injection attacks and cloning through electromagnetic analysis as an RO PUF; thus, the use of the TERO PUF solves these safety problems [13].
- STRO PUF—The principle of operation is based on self-timed ring oscillators (STROs). A challenge generator selects two STROs from each group. The output signal of the oscillators passes through the corresponding multiplexers, and the unpredictable variations in the frequencies of the STROs are captured by using a frequency comparator, which generates the output bit. An STRO is an oscillator built by looping a micropipeline control circuit back to itself. Each stage of the pipeline is a Müller gate and an inverter. Asynchronous communication allows the propagation of several events within the oscillator without colliding. The resulting jitter propagates through each stage of the ring. This design increases the probability of capturing the true randomness from the ring oscillator signal. A properly loaded ring exhibits a special oscillation mode in which all events have the same probability of occurrence [14]. Therefore, an STRO-based PUF can be easily configurable by specifying the number of stages of the rings and selecting the number of circulating events.
- GARO PUF—This is constructed similarly to a linear feedback shift register (LFSR), with a difference in that the memory element is replaced with an inverter. It uses complex XOR feedback that is analogous to the Galois, thus creating Galois ring oscillators (GAROs). It is well known that the oscillators implemented in different locations in an integrated circuit present statistically significant differences [15]. Consequently, a GARO-based PUF compares the value of the bias of the GARO’s state instead of the frequencies of ring oscillators.

The above structures share a common feature—the multiple replications of the oscillator structure to perform a comparison, resulting in a unique system response. Our proposed solution removes the burden of implementing multiple oscillators, as it is based on only one RO that returns a sequence of internal states. The rest of the identifying structure can be transferred to the authorizing device so that a significant part of the authorized device’s resources can be released.

## 3. Proposed Identification Algorithm

The algorithm proposed in this paper establishes a novel class of RO PUFs. The basis of the structure is a Fibonacci ring oscillator, which combines the pseudo-random properties of an LFSR with the true randomness of the oscillation jitter of RO-based random number generators (RNGs). Before reaching sufficient randomness, the applied structure exhibits certain repeatability and uniqueness of the initial sequence of internal states. Therefore, FIRO boot data can be exploited for authentication and authorization. To obtain sufficient identification data, the proposed solution engages the restart method. This approach ensures the reuse of the existing RNG, rather than multiplying the RO in a dedicated structure. Data obtained from several reboots are used to extract a secret key. This innovative algorithm for obtaining the key can be successfully implemented both on the authorized side and on the authorizing side. In the latter, the authorized device only sends a boot data packet to the authorization center where its key is restored. At this point, it is worth noting that careful resource management is of particular importance in the context of the intensive development of an IoT network. Therefore, transferring the responsibility for restoring the keys from randomized data to the authorization center significantly releases the resources of many low-complexity devices.

### 3.1. Fibonacci Ring Oscillator

**Figure 1:**
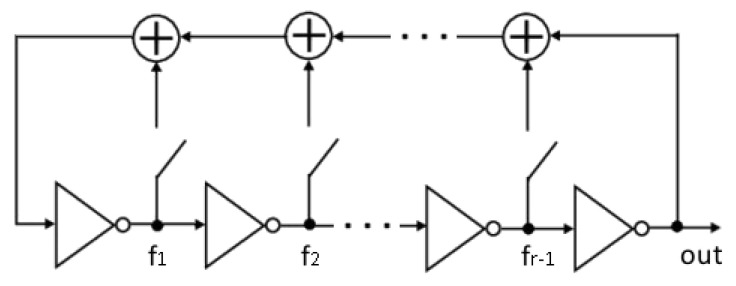
Fibonacci ring oscillator.

The Fibonacci ring oscillator consists of inverters connected in a loop by XOR gates, which define a feedback polynomial (Figure 1). Analogously to the classical LFSR Fibonacci type, but with memory elements replaced with inverters, the feedback polynomial should be primitive. The structure of the FIRO can consist of either an odd or an even number of inverters, except for two, while the output signal can be taken from any inverter’s output [7]. An ideal FIRO exhibits a pseudo-random operation mode for a shift register; however, in a real circuit, random transition times and propagation delays in all paths and gates introduce the true randomness of physical phenomena and production inaccuracies. Therefore, the output of the oscillating signal comprises both pseudo-randomness and true randomness. Moreover, extensive research and implementation of FIRO structures in FPGAs have shown that even when using the same feedback polynomial, the behavior of the system can change drastically depending on the location of the system in the FPGA [16]. Considering the combination of the exhibited pseudo- and true randomness and the unique behavior of each system, the above provides a strong rationale for the identifiability of the FIRO structures necessary for creating a secure PUF.

### 3.2. Restart Method

The restart method was originally designed not to evaluate the randomness contained in the output of a single-ring oscillator, but to assess the influence of deterministic components on the production of consecutive bits [17]. Suppose that, by performing a single restart, the *N*-bit data sequence is produced and *M* restarts of the oscillator are performed. After the experiment, a matrix of size M×N is obtained. If the sequences are a result of nondeterministic phenomena, each restart—with the same initial conditions—produces significantly different strings. Otherwise, if the sequences are created due to deterministic phenomena, the results of the generation will be the same or very similar. In more complex cases that combine both of the above, it is expected that the *N*-element sequences collected should be truly random from a certain $n=n_{min}$, n=1,2,…,N, for which sufficient physical randomness in the system is obtained, e.g., through the accumulation of jitter. The authors of [17] observed that such a situation occurs for relatively large $n_{min}$ for RO-based RNGs.

### 3.3. Common Feature Extraction

**Figure 2:**
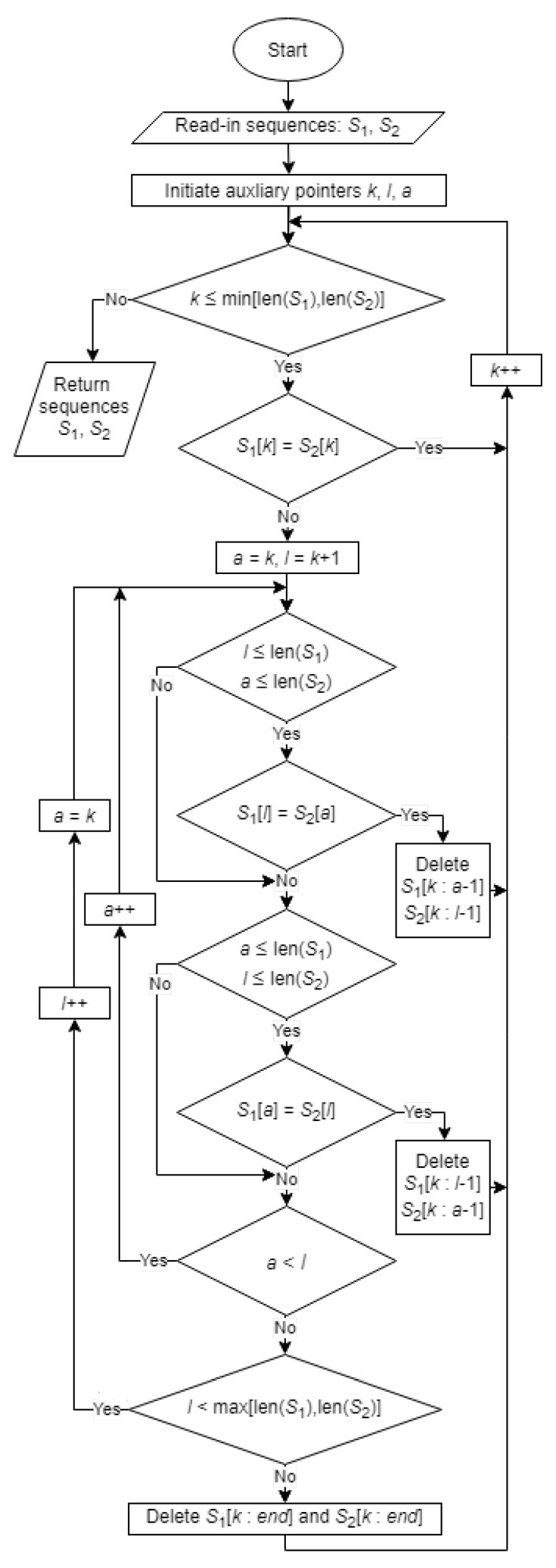
Block diagram of the algorithm for extracting common elements of two random sequences.

Inspired by the effectiveness of the algorithms for sequence alignment, in this paper, we present a new feature extraction algorithm based on a low-complexity sequence comparison. The proposed solution successively searches for common elements in the tested sequences and excludes matchless ones (Figure 2). The algorithm starts by fetching two random binary sequences that are read in as strings of four-bit numbers, i.e., $S_{1}$ and $S_{2}$. Then, for the main rolling index *k*, it successively compares $S_{1}\left[k\right]$ with $S_{2}\left[k\right]$. In the case of a match, it increments the index *k*, but for a mismatch, i.e., when $S_{1}\left[k\right]\neq S_{2}\left[k\right]$, it initializes the auxiliary index l=k+1. The mismatch triggers a search through the sequences by incrementing the *l* index until either an element in the range from *k* to *l* from the opposite sequence that matches $S_{1}\left[l\right]$ or $S_{2}\left[l\right]$ is found or the end of any sequence is reached.

Searching for a match at positions other than $S_{1}\left[k\right]=S_{2}\left[k\right]$ requires the removal of mismatched elements. This requires the creation of an appropriate cost function that should favor an even reduction of both sequences, rather than just one of them. To achieve the intended goal, the proposed algorithm assumes an exponential increase in cost for a series of deletions, i.e., for $d_{i}$ consecutive deletions, the cost is $2^{d_{i}}$. The total cost of reducing both sequences in a single matching attempt is, therefore, $2^{d_{1}}+2^{d_{2}}$. The cost function does not require calculation if it is satisfied by keeping the appropriate order of index checking.

**Figure 3:**
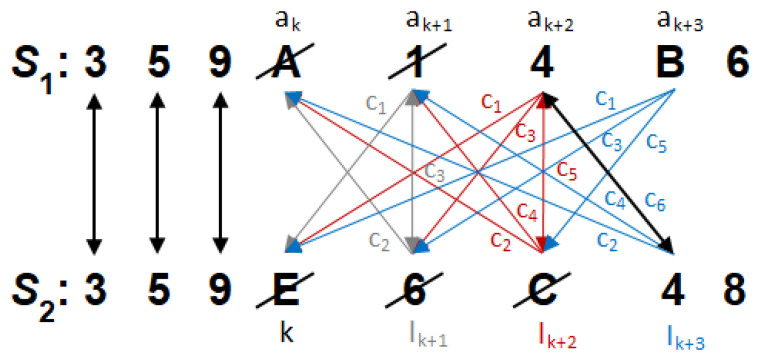
An example of the operation of the algorithm—matching common elements and removing mismatches.

Match optimization is performed by additionally checking if any element of $S_{1}$ matches any element of $S_{2}$ in the current range of optimization defined by the distancing indexes, *k* and *l*. For an increasing auxiliary index *a* starting at position *k* and pointing to the currently compared item, i.e., $S_{1}\left[a\right]$ or $S_{2}\left[a\right]$, a match with the current reference is sought, i.e., $S_{2}\left[l\right]$ and $S_{1}\left[l\right]$, respectively. The element found with the lowest possible index *a* preceding *l* corresponds to the minimization of the reduction cost. An example of the algorithm’s operation is shown in Figure 3.

After reaching a mismatched pair in the *k*-th position, i.e., A≠E, the algorithm begins searching for the following similarities to ensure the lowest cost. Each *i*-th successive search iteration covers $c_{j}$ sequential checks of matching $S_{2,1}\left[a\right]$ to the current reference $S_{1,2}\left[l_{k+i}\right]$, where *a* sweeps $\langle k,l_{k+i}\rangle$ and j∈〈1,2i+1〉. The first match that is found to satisfy the minimum cost condition aborts the search and triggers the removal of previous mismatched elements from both sequences.

Note that as a consequence of continually reducing the sequences, their lengths may not be equal. The algorithm is complemented by boundary conditions to ensure correct searching in the case of strings of different lengths.

### 3.4. Construction of an FIRO-PUF

**Figure 4:**
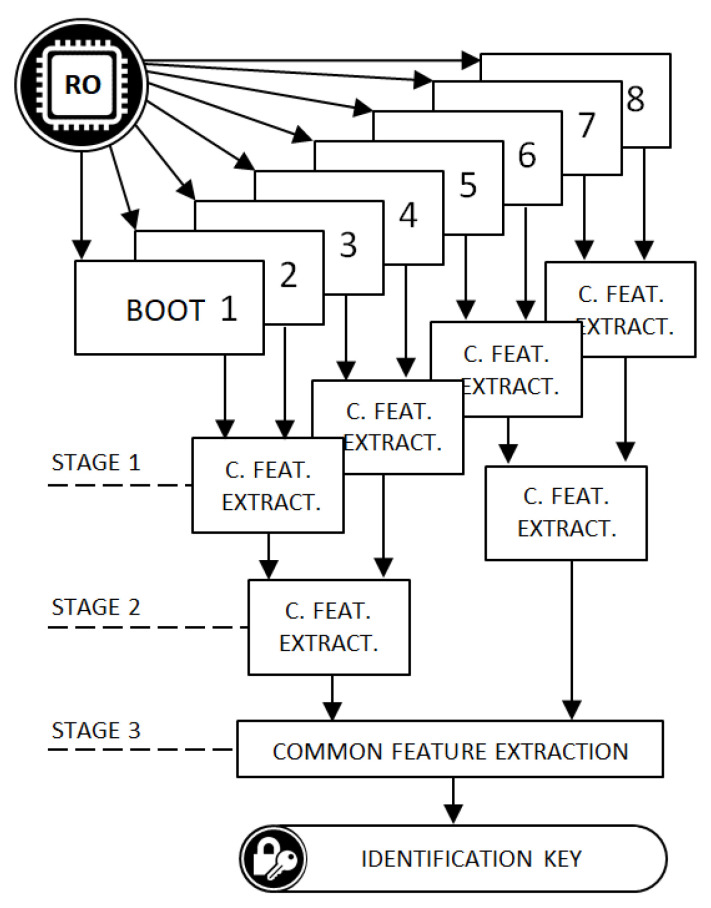
Boot-set data processing for obtaining the identification key in the tree-structured process of the extraction of multiple common features.

The proposed methodology for obtaining the identification key assumes that the authorized device is equipped with an FIRO that consists of four inverters, thus realizing a primary polynomial. During a single FIRO boot, 128 consecutive internal states are collected in the form of four-bit numbers composed of the current outputs of the inverters. The requisite reboot dataset requires eight restarts that return a sequence of 512 bits each. The collected data are subjected to a three-stage extraction of common functions, which are carried in disjoint pairs according to the scheme shown in Figure 4. The application of this tree-structured data processing suppresses the randomness resulting from the system’s operating conditions and extracts from the randomized data a repeatable sequence with randomness that results only from post-production inaccuracies of the device, i.e., a unique authorization key.

## 4. Experimental Results and Discussion

**Figure 5:**
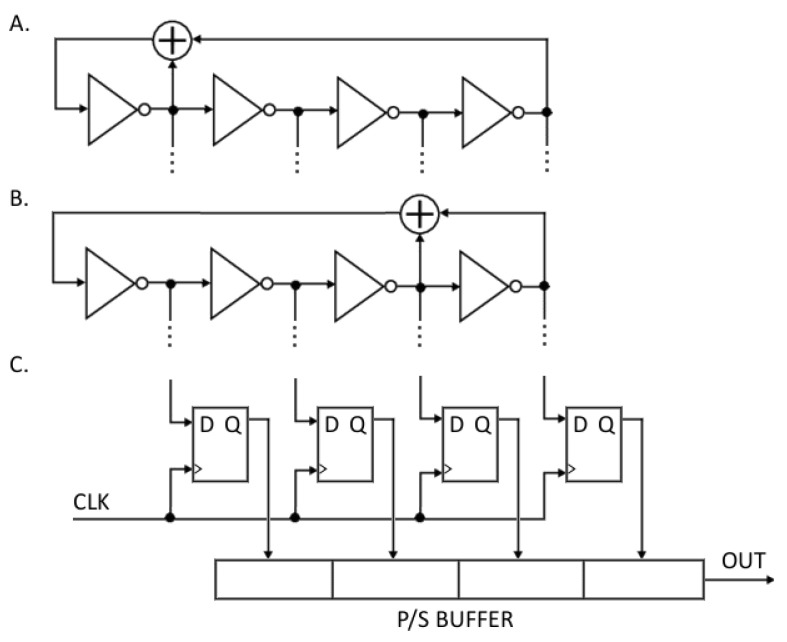
Experimental FPGA setup. (**A**) $x^{4}+x+1$ FIRO, (**B**) $x^{4}+x^{3}+1$ FIRO, (**C**) internal FIRO state sampler.

**Figure 6:**
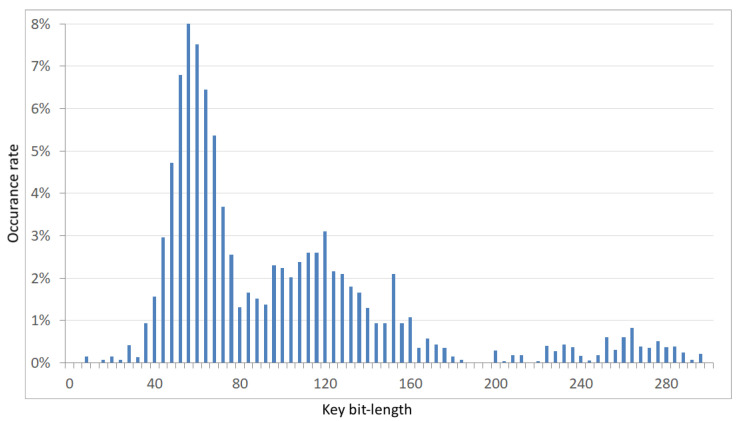
Histogram of the keys’ bit-lengths after a three-stage reduction of the initial boot-set of eight 512-bit random sequences.

In this research, we used twelve evaluation boards from two major FPGA manufacturers, i.e., Xilinx and Intel. From Xilinx, we used six boards with a Spartan 6 device, and from Intel, we used six boards with MAX10. The selection of the FPGAs from the two different manufacturers was intended to test the portability and practicality of the proposed solution for various inaccuracies in separate production processes. Two FIRO-PUF generators were implemented with the most popular four-order primitive polynomials. The first was $x^{4}+x+1$ and the second was $x^{4}+x^{3}+1$ (Figure 5). The clock sampling in the internal states was set to 100 MHz. After buffering, the data collected in the sampling process were sent to the computer, which acted as an authorizing center. Eighty restarts were performed with the same initial conditions for both polynomials in each evaluation board from both sets. To create the tree structure, which is shown in Figure 4, the data collected from the reboots for each polynomial and board were grouped by eight into overlapping sets as follows: The data from the first eight restarts formed the first group, second group contained the data from the second to the ninth restart, the third group contained the data from the third to the tenth restart, and so on. As a result, seventy-two groups were formed. Then, the common feature extraction algorithm was used on each group (Figure 4). The outcome of the algorithm was a single device identification key. Seventy-two keys were obtained for each board and polynomial. The empirical distribution of the effective bit-length of the obtained keys is shown in the Figure 6. Each key extraction step reduced the input string by approximately half its length. On average, after a three-stage reduction of the initial boot-set of eight 512-bit random sequences, a 100-bit key was extracted. It should be noted at this point that the length of the obtained key may be extended. As mentioned in Section 3.2, the proposed solution is based on the number of initial weakly random bits, which, in the experiment conducted here, were collected at a frequency of 100 MHz, asynchronously with the frequency of the ring oscillator. As shown by the authors of [18], for a ring with four elements, the delay in the state transition frequency was 645 MHz, so the number of bits collected to generate the key could even be increased by six times.

**Figure 7:**
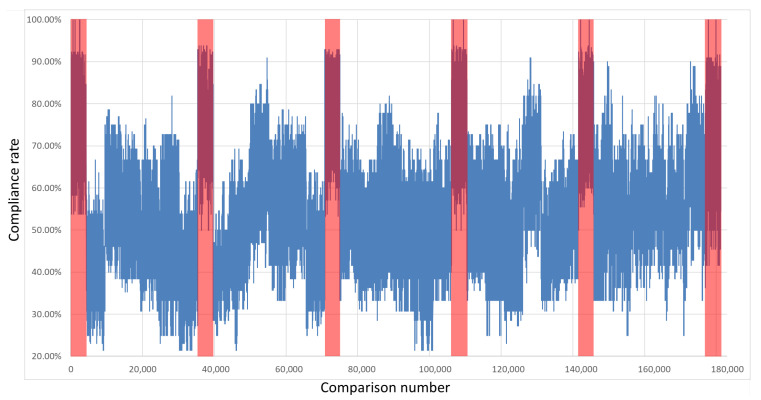
Results of the comparison of each key pair for all boards in a set—Xilinx Spartan 6 $x^{4}+x+1$.

**Figure 8:**
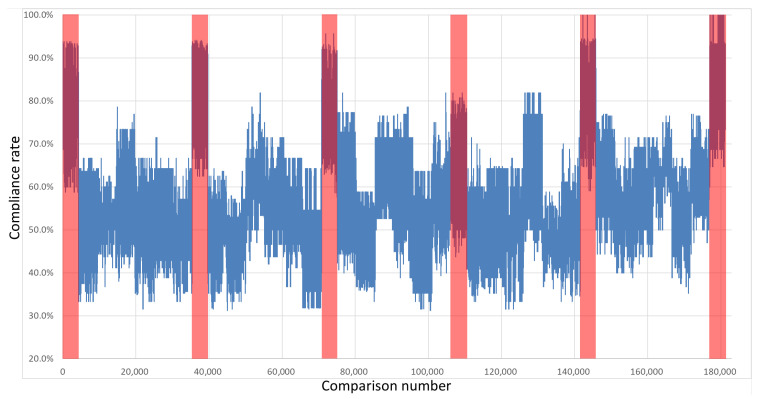
Results of the comparison of each key pair for all boards in a set—Xilinx Spartan 6 $x^{4}+x^{3}+1$.

**Figure 9:**
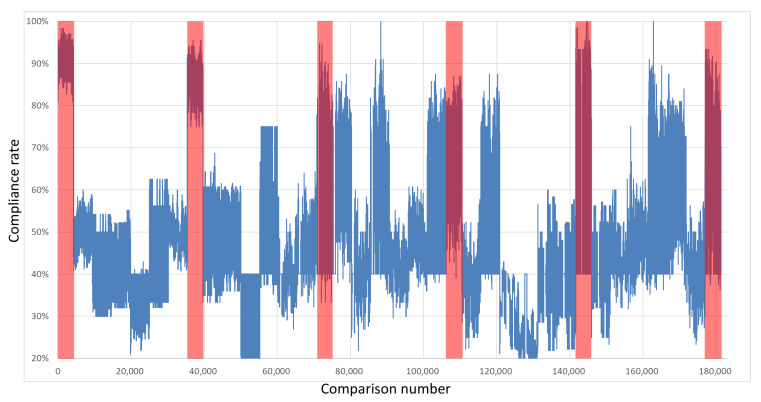
Results of the comparison of each key pair for all boards in a set—Intel MAX10 $x^{4}+x+1$.

**Figure 10:**
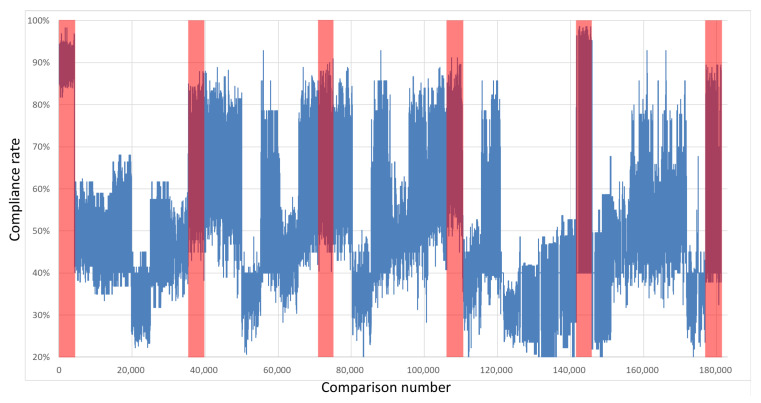
Results of the comparison of each key pair for all boards in a set—Intel MAX10 $x^{4}+x^{3}+1$.

The keys were compared with each other to test their compatibility. Importantly, the comparison was performed with the exclusion of every pair of keys derived from overlapping restart groups from the same integrated circuit with the same polynomial. Therefore, each board was described with 4291 own-key comparisons and 25,920 comparisons with keys sourced from other boards. Figure 7, Figure 8, Figure 9 and Figure 10 show the percentages of compatibility for comparisons of each pair of keys from the specified board set and polynomial. On the *x*-axis, the numbers of comparisons are organized as follows: from 0 to 4290, keys from board B1 are compared with other keys from the same board; from number 4291 to 9474, keys from board B1 are compared with keys from board B2; from 9475 to 14,659, keys from board B1 are compared with keys from board B3, etc. Analogously, for board B2, the comparisons from 30,211 to 35,394 cover keys from board B2 in comparison with keys from board B1; then, for the next 4291 comparisons, the keys from B2 are compared internally, followed by comparisons with the remaining four boards and so on until 176,975 to 181,265, where keys from board B6 are compared with one another.

The regions marked in red cover the results of the comparisons between the same boards. The first region marked in red represents the comparison of keys from board B1; analogously, the second red region marks keys from board 2 that were compared with each other, and so on. It can be seen that in these regions, the percentage of congruence is higher than for the rest of the results, which means that by using the proposed common feature extraction algorithm, it is possible to obtain the identifiability of specific integrated circuits with respect to other circuits of the same series.

**Figure 11:**
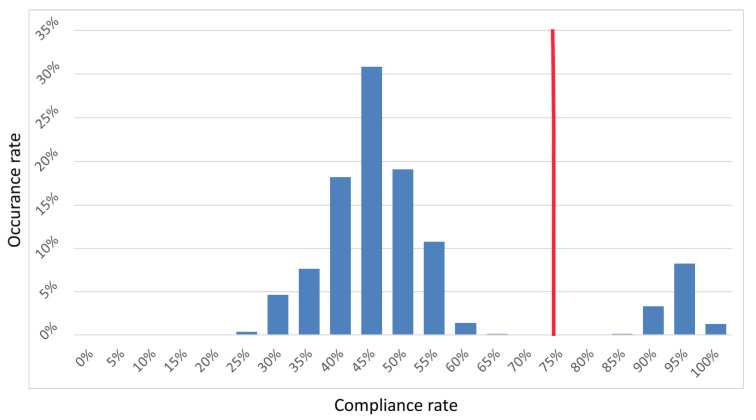
Histogram of the keys’ compliance with two distinguishable normal sub-distributions.

To correctly identify the source board of each received key, a histogram of the compliance of individual keys from a given board with keys from other boards was made. Figure 11 shows the empirical results—a mixed distribution with two clearly distinguishable normal sub-distributions. The first had a mean value of 45% and the second had a mean value of 95%. The spacing between the distributions reached the center at the 75% point, which was taken as the decision threshold. If the result of the comparison was lower than or equal to a 75% match, the keys came from different boards. If it was greater, the keys came from the same FPGA circuit.

The results in Table 1, Table 2, Table 3 and Table 4 show the percentages of keys whose compatibility was greater than 75% with all keys when comparing keys from specified board sets and polynomials. The labels of the columns and rows show the numbers of the boards. Intersections of the same labels indicate the results of the comparisons of keys derived from the same FIRO circuits.

It can be seen that the compliance percentage when comparing the same boards was high and varied from 62.24% in the worst case to 100%, i.e., when all keys matched. On average, a compatibility ratio of 85% was achieved. However, when comparing different boards, the compliance ratio was very small. In the worst case, it reached 6.21%, but more often, it was 0%, resulting in 0.5% on average. Moreover, the results were distinguishable according to the manufacturers. Implementation in Xilinx devices provided a lower deviation of the results, reducing it by 3/4 compared to that found in the Intel circuits. The study of the latter, however, introduced significant efficiency limits into the analysis of the proposed solution. The uniqueness and repeatability of the keys generated from the Intel MAX10 B1 boot-set represented the highest achievable performance, while the specificity of the B4 board showed its limited usability and scope for further improvements. The differences in performance between devices from different manufacturers resulted directly from the variances in the production process. Higher manufacturing inaccuracies amplify the non-deterministic behavior of a ring oscillator and, thus, reduce the repeatability of the obtained key. Overall, the above results exhibit a high degree of identifiability of the FPGAs with the proposed implementation of an FIRO-PUF and a satisfactory degree of unclonability. It is also worth noting that the systems in which the polynomial $x^{4}+x^{3}+1$ was implemented achieved the compliance results that were, on average, lower by 5% than for the polynomial $x^{4}+x+1$. The observed difference was due to the disproportion in the path lengths propagating the signal to the inputs of the XOR gate (Figure 5). The longer propagation difference resulted in a more frequent appearance of a metastable state at the output. This resulted in a faster accumulation of jitter and, hence, a greater variance of the values at the system output.

## 5. Conclusions

This article presents a new class of physical unclonable functions based on the Fibonacci ring oscillator and an innovative method for extracting an identification key from an oscillator boot-set. An analysis of the compatibility of the keys produced by the developed function showed that the proposed solution was, on average, characterized by a high degree of identifiability (85%) with a low misidentification rate (0.5%). The obtained results also showed a high degree of unclonability, i.e., the same functions implemented in different FPGAs of the same series returned significantly different answers (keys). An important feature of the solution presented here is the simple structure of the basic oscillator and the simplicity of the feature extraction algorithm, which is based on iterations, increments, and comparisons. This provides a possibility for easy realization of hardware as built-in blocks in FPGAs and ASICs. It can be successfully used in systems that require the generation of keys that enable the identification of a given object, especially in simple cryptographic systems, e.g., in IoT systems.

## Figures and Tables

**Table 1:** Compatibility ratio for the Xilinx Spartan 6 $x^{4}+x+1$.

	B1	B2	B3	B4	B5	B6
B1	91.82%	0%	0%	0.17%	0%	0%
B2	0%	85.06%	0%	0%	1.66%	0%
B3	0%	0%	91.12%	0.46%	0%	0.21%
B4	0.17%	0%	0.46%	86.95%	0%	0%
B5	0%	2.39%	0%	0%	83.45%	0.48%
B6	0%	0%	0.21%	0%	0.5%	88.55%

**Table 2:** Compatibility ratio for the Xilinx Spartan 6 $x^{4}+x^{3}+1$.

	B1	B2	B3	B4	B5	B6
B1	73.75%	0%	0.14%	0%	0.02%	0.02%
B2	0%	75.57%	0%	0%	6.19%	0.1%
B3	0.12%	0%	81.33%	0.08%	0%	0.44%
B4	0%	0%	0.08%	72%	0%	0%
B5	0.02%	6.21%	0%	0%	78.83%	1.87%
B6	0.02%	0.1%	0.44%	0.21%	1.87%	83.31%

**Table 3:** Compatibility ratio for the Intel MAX10 $x^{4}+x+1$.

	B1	B2	B3	B4	B5	B6
B1	100%	0%	0%	0%	0%	0%
B2	0%	99.81%	0%	0%	0%	0%
B3	0%	0%	70.16%	3.84%	0%	5.17%
B4	0%	0%	4.26%	78.48%	0%	1.47%
B5	0%	0%	0%	0%	76.83%	0%
B6	0%	0%	5.4%	1.66%	0%	74.76%

**Table 4:** Compatibility ratio for the Intel MAX10 $x^{4}+x^{3}+1$.

	B1	B2	B3	B4	B5	B6
B1	100%	0%	0%	0%	0%	0%
B2	0%	72.73%	2.08%	4.68%	0%	1.58%
B3	0%	1.49%	88.37%	1.7%	0%	2.04%
B4	0%	5.29%	1.66%	62.24%	0%	1.96%
B5	0%	0%	0%	0%	75.99%	0%
B6	0%	1.74%	2.51%	1.91%	0%	62.63%

## Data Availability

The data that support the findings of this study are available from the corresponding authors, Ł.M. and J.N., upon reasonable request.

## References

[1] Babaei A., Schiele G. (2019). Physical Unclonable Functions in the Internet of Things: State of the Art and Open Challenges. Sensors.

[2] Garcia-Bosque M., Diez-Senoras G., Sanchez-Azqueta C., Celma S. Introduction to phisically unclonable functions: Properties and applications. Proceedings of the 24th European Conference on Circuit Theory Design (ECCTD).

[3] Adames I., Das J., Bhanja S. Survey of emerging technology based physical unclonable functions. Proceedings of the 2016 International Great Lakes Symposium on VLSI (GLSVLSI).

[4] Shamsoshoara A., Korenda A., Afghah F., Zeadally S. (2020). A survey on physical unclonable function (PUF)-based security solutions for Internet of Things. Comput. Netw..

[5] Guajardo J., van Tilborg H., Jajodia S. (2011). Physical Unclonable Functions (PUFs). Encyclopedia of Cryptography and Security.

[6] Rührmair U., Sehnke F., Sölter J., Dror G., Devadas S., Schmidhuber J. Modeling attacks on physical unclonable functions. Proceedings of the 17th ACM conference on Computer and communications security (CCS ’10).

[7] Dichtl M., Golic J.D. High speed true random number generation with logic gates only. Proceedings of the Conference on Cryptographic Hardware and Embedded Systems (CHES 2007).

[8] Jessa M., Matuszewski L. Enhancing the Randomness of a Combined True Random Number Generator Based on the Ring Oscillator Sampling Method. Proceedings of the 2011 International Conference on Reconfigurable Computing and FPGAs.

[9] Avaroglu E. (2020). The implementation of ring oscillator based PUF designs in Field Programmable Gate Arrays using of different challenge. Phys. A Stat. Mech. Appl..

[10] Sakhare S., Sakhare D., Merchant S., Warhade K., Adhikari D. (2021). Ring Oscillator-Based Physical Unclonable Functions. Advances in Signal and Data Processing.

[11] Suh E., Devadas S. Physical unclonable functions for device authentication and secret key generation. Proceedings of the 44th Annual Design Automation Conference (DAC’07).

[12] Sahoo S., Kumar K., Mahapatra K. (2017). A novel current controlled configurable RO PUF with improved security metrics. Integration.

[13] Marchand C., Bossuet L., Mureddu U., Bochard N., Cherkaoui A., Fischer V. (2017). Implementation and Characterization of a Physical Unclonable Function for IoT: A Case Study With the TERO-PUF. IEEE Trans. Comput. Aided Des. Integr. Circuits Syst..

[14] Gimenez G., Cherkaoui A., Fesquet L. A Self-Timed Ring based PUF. Proceedings of the 26th IEEE International Symposium on Asynchronous Circuits and Systems (ASYNC).

[15] Garcia-Bosque M., Diez-Senoras G., Sanchez-Azqueta C., Celma S. (2020). Proposal and Analysis of a Novel Class of PUFs Based on Galois Ring Oscillators. IEEE Access.

[16] Addabbo T., Fort A., Moretti R., Mugnaini M., Vignoli V., Bosque M. Lightweight true random bit generators in PLDs: Figures of merit and performance comparison. Proceedings of the IEEE International Symposium on Circuits and Systems (ISCAS).

[17] Jessa M., Jaworski M. Randomness of a combined TRNG based on the ring oscillator sampling method. Proceedings of the ICSES 2010 International Conference on Signals and Electronic Circuits.

[18] Jessa M., Matuszewski L. The use of delay lines in a ring-oscillator-based combined true random number generator. Proceedings of the International Conference on Signals and Electronic Systems (ICSES).

